# Instagram Content Addressing Pruritic Urticarial Papules and Plaques of Pregnancy: Observational Study

**DOI:** 10.2196/26200

**Published:** 2021-02-11

**Authors:** Ashley Payton, Benjamin K P Woo

**Affiliations:** 1 Department of Psychiatry Olive View Medical Center University of California Los Angeles Medical Center Sylmar, CA United States; 2 College of Osteopathic Medicine of the Pacific Western University of Health Sciences Pomona, CA United States

**Keywords:** pruritic urticarial papules and plaques of pregnancy, dermatology, rash, pregnancy, obstetrics, dermatosis, Instagram, social media, patient education

## Abstract

**Background:**

Pruritic urticarial papules and plaques of pregnancy (PUPPP) is the most commonly diagnosed pregnancy-specific dermatosis. It presents with intense pruritus and can be difficult to manage, which encourages mothers to look to social media for camaraderie and advice.

**Objective:**

This study aimed to characterize the sources and thematic content of Instagram posts in order to define influential groups of users. Our goal was to determine the status of online discourse surrounding PUPPP and elucidate any potential space for health care provider intervention via creation of Instagram accounts dedicated to information dissemination for patient populations.

**Methods:**

Three hashtag categories were selected (#PUPPP, #PUPPPs, and #PUPPPrash), and the top public posts from each were analyzed and organized by source and by thematic content. The numbers of likes and comments were also recorded.

**Results:**

Among the top 150 posts in each hashtag category, only 428 posts in total were eligible for this analysis. Majority (316/428, 73.8%) of posts were created by mothers who experienced PUPPP. These posts were testimonial accounts in nature. A small fraction of posts (14/428, 3.3%) were generated by physician accounts. Posts from blogs with extensive followings garnered the most attention in the form of likes and comments.

**Conclusions:**

Mothers experiencing PUPPP comprised the majority of accounts posting under the hashtags selected. The most common themes included pictures of the rash and personal testimonies. Posts under blog posts received the most likes and comments on average. There is space for physician and health care specialists to improve their social media presence when it comes to discourse surrounding PUPPP. Patients are seeking out communities on social media, like Instagram, in order to have questions answered and obtain advice on management. Accounts with large followings tend to have more likes and more comments, which encourages information dissemination and awareness. Thus, we suggest that physicians create content and potentially partner with blog-type accounts to improve outreach.

## Introduction

Pruritic urticarial papules and plaques of pregnancy (PUPPP) is the most common pregnancy-specific dermatosis affecting about 1 in 200 pregnancies [1]. It is also known as polymorphic eruption of pregnancy. PUPPP is more common in primiparous women and is characterized by erythematous papular lesions that classically arise within the confines of striae distensae on the gravid abdomen [1,2].

Typically, the eruptions begin on the abdomen and can spread to the thighs, arms, and buttocks, with onset occurring typically in the third trimester [3,4]. Symptoms tend to resolve 7 to 10 days postpartum [4]. However, this rash can be very pruritic, extensive, and difficult to manage for patients, especially in multiple gestation cases [4]. Many mothers try antipruritus creams and medications, with little or short-lasting relief. This could be one factor driving mothers with PUPPP to seek support in various outlets, including social media.

Social media has taken the spotlight in recent years as a tool for human interaction, which has changed how we learn from and engage with peers. Particularly within younger generations that grew up with internet access, it is increasingly common to find that people turn to social media for information and advice. A recent survey found that 72% of people reported turning to the internet to look up health information within the last year [5]. The convenience of the internet at the tips of our fingers has made it a preferred source for many Americans searching for answers.

Social media has become a way for patients with various unique conditions to post and find camaraderie with others who have similar afflictions, including during pregnancy [6]. We must be cognizant of the power that social media has to influence our decision-making ability in this regard [7,8]. Endorsements on social media sites, such as Instagram, have been shown to activate reward centers of the brain, making social media a powerful tool for peer influence [8]. According to Instagram’s webpage, it boasts of having over 1 billion users worldwide. The Pew Research Center generated an estimate that roughly 72% of American adults have at least one social media account and that 37% have an Instagram account as of 2019 [9]. Creating posts for Instagram is free, and posts can be made available to the public. This makes the app a cost-effective and efficient way for health care specialists to widely distribute quick medical information to the public at large. In light of this, it is important to analyze how patient populations interact with content on social media so that we can determine whether there is space for health care professionals to provide evidence-based medical information and quell patient skepticism about information they are finding online.

PUPPP is a lesser publicized affliction, and thus, its discussion is not common in the public arena despite its relatively high prevalence in pregnant and postpartum mothers [1]. The rash can dramatically impact mothers during pregnancy and alter their experience. We hope to be able to shed some light on what information is being distributed on popular social media sites. In this study, we seek to characterize posts regarding PUPPP circulated on Instagram from the public. It is our goal to determine what discourse is generated by and for these pregnant mothers in order to define a potential space for increased physician and health care provider intervention, education, and advocacy.

## Methods

### Data Collection

Using the Instagram app, hashtag-based key terms were searched and identified (n=3; #PUPPP, #PUPPPs, and #PUPPPrash). Note that capitalization does not make a difference on the app hashtag search function; thus, “#PUPPP” yields the same result as “#puppp.”

The top 150 posts from each tag were selected for analysis from all public posts. To be included in the study, the picture’s caption had to include information or opinions regarding PUPPP. We excluded posts that were considered private because they would not be readily accessible to the public when using Instagram’s search function. We excluded posts that had irrelevant material (ie, posts about puppies that were tagged within the #puppp thread and posts that did not include content regarding PUPPP) or were repeat tags (ie, posts tagged in the #puppp and #puppps categories). With these criteria, two of the tag categories yielded fewer than 150 posts that qualified for the study.

### Data Analysis

Each post was assigned exclusively to a category based on source. The categories included the following: (1) mother, (2) physician/health care provider, (3) health care organization, (4) company/product, and (5) blog/blogger (Table 1). For further clarification, the category for physician/health care provider was scrutinized even further to determine if posts were from physicians or other providers such as midwives and doulas. In order for a post to be determined to be from a mother, caption information was taken into account and designation was granted if first person language was used. Many of these posts were accompanied by “selfies” that contributed to the decision of assigning a post to the mother category.

Characterization of the thematic content of each post was then determined by the team. Thematic content was categorized nonexclusively, meaning that each post could be assigned to more than one category based on image content as well as accompanying caption content. These categories included the following: (1) testimony, (2) educational information, (3) picture of PUPPP rash, (4) therapy advice & guidance, (5) blog post, and (6) product promotion (Table 1).

**Table 1:** Stratification methodology of Instagram posts that met the inclusion criteria.

Post source categories (exclusive assignment)^a^	Thematic content categories (nonexclusive assignment)^a^
1. Mother	1. Testimony
2. Physician/health care provider	2. Educational information
3. Health care organization	3. Picture of PUPPP^b^ rash
4. Company/product	4. Therapy advice & guidance
5. Blog/blogger	5. Blog post
	6. Production promotion

^a^Individual posts could be placed exclusively in one category based on their source but were nonexclusively categorized by content of the post.

^b^PUPPP: pruritic urticarial papules and plaques of pregnancy.

The numbers of comments and endorsements or “likes” were recorded for each post after the characterization process. The average numbers of likes and comments were then calculated within each tag category.

## Results

### Tags

As of November 23, 2020, there were 2100 posts tagged with #PUPPP, 599 posts tagged with #PUPPPs, and 189 posts tagged with #PUPPPrash, which were publicly available on Instagram (totaling 2888 posts).

After determining which posts were eligible for study, 150 were included under #PUPPP, 141 were included under #PUPPPs, and 137 were included under #PUPPPrash (totaling 428 posts).

### Source Categorization

Posts in all three tags were placed into one of five designated categories for characterization of the source. Overall, we found that the majority of posts available were created and shared by those who identified themselves as mothers (316/428, 73.8%), followed by blogs (58/428, 13.6%), companies (28/428, 6.5%), physicians or health care providers (14/428, 3.3%), and health care organizations (12/428, 2.8%) (Table 2).

**Table 2:** Post source categorization.

Tag category	Post source, n (%)				
	Mothers	Health care organizations	Physicians and health care providers	Companies	Blogs
#PUPPP (N=150)	134 (89.3%)	9 (6.0%)	7 (4.7%)	0 (0%)	0 (0%)
#PUPPPs (N=141)	132 (93.6%)	1 (0.7%)	0 (0%)	8 (5.7%)	0 (0%)
#PUPPPrash (N=137)	50 (36.5%)	2 (1.5%)	7 (5.1%)	20 (14.6%)	58 (42.3%)
Overall (N=428)	316 (73.8%)	12 (2.8%)	14 (3.3%)	28 (6.5%)	58 (13.6%)

In all categories, except for #PUPPPrash, mothers themselves were the predominant posters of content regarding PUPPP. In #PUPPPrash, the largest portion of content and discussion involved blogs (58/137, 42.3%), most of which were identified as “maternity lifestyle blogs” where women share experiences, advice, and information regarding pregnancy and motherhood to their followers (Table 2).

Only 14 posts came from health care providers overall, seven of which were from physicians licensed with an MD (Doctorate in Medicine) or DO (Doctorate in Osteopathic Medicine) medical degree (Table 2). The other seven advertised themselves as mid-level providers, such as nurse practitioners, lactation consultants or midwives, and doulas. This content made up 3.3% (14/428) of the overall number of posts.

#PUPPP had the majority of posts from accounts deemed as “health care organizations,” such as a public account, @skincancerderminstitute, a dermatology clinic. Nine of the 12 posts coming from health care organizations were in this tag group. Other organizations represented were centered on pregnancy and women’s health.

The “companies” category, which we defined as any account tied to a business that advertised a product or service that they themselves sell and/or provide for financial gain, was most prominent in the #PUPPPrash category, comprising 14.6% (20/137) of all posts analyzed (Table 2). Of the 28 posts from companies, 20 were found under #PUPPPrash.

### Thematic Content Analysis

All posts were categorized nonexclusively into six categories based on the content in the image or the caption associated with the image.

By and large, the category “testimony” comprised a majority of the posts across all three tag groups. Out of all 428 posts, 309 (72.2%) were classified as a “testimony” based on the content within the caption provided by the poster. This meant that 72.2% of all posts contained personal accounts and anecdotes from mothers who had experienced PUPPP during one or more of their pregnancies (Table 3). Most of these testimonies were mothers describing their journeys, expressing frustration with the pruritic rash, and providing encouragement to their followers who may be experiencing the same affliction.

**Table 3:** Analysis of thematic content of posts in each tag category and overall (N=428).

Theme^a^	Tag category			
	#PUPPP, n	#PUPPPs, n	#PUPPPrash, n	Across all tags, n (%)
Testimony	91	94	124	309 (72.2%)
Education	20	8	1	29 (6.8%)
Therapy advice	10	14	5	29 (6.8%)
Blog post	0	11	89	100 (23.4%)
Production promotion	15	23	5	43 (10.0%)
Picture of rash	58	55	115	228 (53.3%)

^a^Posts were nonexclusively categorized, that is, each post could be tallied in more than one of the six themes represented.

Posts were given a designation under the category “education” if they provided objective and factual medical information about PUPPP. Posts in this category, for example, included infographics, diagrams, and other texts that would provide information to moms about what PUPPP is, what the symptoms are, and what standard treatment includes, and/or provide epidemiological information. A popular fact included frequently in educational posts was that about 1 in 150 to 200 women will be affected by PUPPP [1,3].

Overall, 228 (53.3%) posts contained a picture of a rash directly. Pictures of a rash included an exposed abdomen with signs of PUPPP. Within the PUPPPrash tag, one picture of one blogger’s rash was reposted 78 times with a copied caption. In the PUPPP and PUPPPs groups, pictures were all personal, meaning they were of the user’s own rash.

Another important finding was that 100 of the 428 (23.4%) posts were from an account that advertised being a blog (Table 3). Blog posts either came from blog accounts, such as accounts that advertised being a “personal” or “maternity” blog, or from mothers who were self-promoting their own personal blogs. Of these 100 posts, 89 were found under the PUPPPrash tag.

Therapy advice included posts in which accounts offered advice on which over-the-counter products or home remedies worked best for the poster’s PUPPP. These posts only comprised 6.8% (n=29) of all posts.

Lastly, product promotion included posts in which a specific product or service was advertised for financial gain by the poster. These posts included products, such as herbal soap bars, creams, and essential oils, designed to help with the symptoms of PUPPP. These posts could have links to the vendor’s Instagram page or website for consumers to purchase products directly. Product promotion posts comprised 10.0% (43/428) of all posts and were more represented in the PUPPPs group followed by the PUPPP group (Table 3).

### Endorsement and Follower Interaction

The number of likes was recorded for each post during analysis, and the average was calculated for each subsection. The highest average number of likes was found in the PUPPPrash tag with 2371.52. The same category also resulted in the highest average number of comments of 34.34 (Table 4).

**Table 4:** Likes and comments broken down by tag category.

Tag category	Mean number of endorsements	Range for the number of likes	Mean number of comments	Range for the number of comments
#PUPPP	50.53	0-458	10.46	0-65
#PUPPPs	67.39	1-1452	8.57	0-46
#PUPPPrash	2371.52	4-38,350	34.34	0-389

The PUPPPrash group had the highest average number of likes but also had the largest range of likes from 4 to 38,350. This category had 10 posts with more than 10,000 likes and 32 posts with between 1000 and 9999 likes. No other category had a post with more than 10,000 likes. PUPPPrash also had the largest average number of comments per post with a range of 0 to 389 (Table 4).

Of note, the PUPPPrash category also had the highest concentration of blog accounts (Table 3). These accounts tended to have more followers, which may have accounted for the higher number of likes and comments per post.

## Discussion

### Principal Findings

The presence of PUPPP on the social media app Instagram is significant yet small in comparison to the estimated number of pregnancies affected each year. With a little under 3000 posts available to the public on the popular app and around 3.7 million births in the United States annually, there appears to be a gap in discourse surrounding this common dermatosis [10]. A search on the Instagram interface reveals that other sequelae of pregnancy, such as hyperemesis gravidarum, have more dedicated posts. For example, searching #hyperemesisgravidarum on Instagram returned approximately 49,200 posts as of November 30, 2020. Despite the low census of posts for PUPPP, the posts included in this analysis represented a diverse pool of sources as well as themes.

Interestingly, the overwhelming majority of posts came from mothers who were affected by the rash. These posts tended to contain testimonial captions and frequently included pictures of the mother’s own rash. Based on caption analysis, most of these testimonial posts were intended to bring awareness to a condition that is considered “embarrassing” by many moms. Posts would include candid accounts of the mothers’ experiences with combating PUPPP. Posts like this help to normalize the discourse and make others feel more comfortable discussing their rash with their followers. Some moms even included pictures of their exposed rash. These vulnerable pictures could put others at ease if the rash looks similar to their own. Overall, these testimonial posts really highlight the community’s honesty with PUPPP and willingness to share their experiences for the benefit of others.

The amount of posts coming from health care professionals made up a small fraction of the sample (14/428, 3.3%). As a potential space for physicians to impact the public beyond their clinical domain, this analysis has made it apparent that there is room for improvement on the part of physicians to guide online discussion. Only 14 of all 428 posts were from professional health care providers, and only seven of those were from physicians (Table 2). A post that is created by a trained medical professional might be viewed as more credible by the public than a post from a layperson, meaning these posts could be more influential. The posts that did come from these accounts tended to be educational in nature with the goal of teaching followers about PUPPP. Again, only a small percentage of posts offered educational material signaling a place for growth for specialists who see and treat patients with PUPPP.

The posts that this study found to garner the most attention, in the form of likes and comments, were blog posts, although they made up only 23.4% (100/428) of the posts across all three investigated tags. Some of these posts gained tens of thousands of likes and hundreds of comments. The blog accounts that many of these “high-earning” posts came from boasted large followings, which may have been a contributing factor for the greater interaction of these posts than posts from any one mother. This makes sense given that Instagram’s algorithm puts pictures from accounts you follow into one’s home feed. Therefore, the more followers an account has, the more interaction its posts will receive. Many of the blogs that earned the most likes were advertised as maternity blogs, and the posts dealt with topics and issues for expecting mothers as well as new mothers. It is possible that mothers who have experienced complications during their pregnancy seek out and follow pregnancy-specific blogs on Instagram in the hope of finding camaraderie and reassurance in their peers and influential users.

### Limitations and Future Directions

Several limitations became apparent during our analysis. First, Instagram has introduced a new policy that does not allow the general public to view all available posts. Currently, there is a disclaimer when one searches any given hashtag that states not all of the most recent posts will show up on the content feed. This is part of a new initiative by social media companies to stop the spread of misinformation.

On Instagram, we are not able to definitively determine the age or gender of the person posting the content as this information is not distributed by each user’s account. This limited our analysis particularly when looking at the posts coming from mothers affected by PUPPP. Access to the demographic information of the mothers would have given us a better idea of what audience characteristics are better represented on Instagram.

There is future potential for a similar study to analyze the content of the comment sections. Previous studies have demonstrated that examining user comments on social media can provide an in-depth view of questions and concerns brought up by patients [11,12]. Characterization of the comments from followers in order to discover the nature of supportive or inquisitive feedback under each post could strengthen the argument that patients are seeking quality medical information on Instagram. A dive into the nuances of conversation between followers and posters could further elucidate the exact needs and curiosities of patients opting to research conditions on social media.

Other studies could be designed with this paradigm in order to examine other lesser known medical conditions in all specialties. It could be interesting to investigate some more “taboo” conditions as well in order to see how willing online users would be to ask about them behind the comfort and convenience of the keyboard rather than in person to their physician.

### Clinical Applications and Conclusions

This study demonstrated that there is a considerable presence on Instagram of the most common dermatosis specific to pregnancy, PUPPP. We were able to examine this common skin condition of pregnancy through the unique lens of publicly available Instagram content. Through the use of hashtags on the popular social media app, we found that mothers with PUPPP readily expressed their experiences, asked questions, and shared advice with their followers. At times, these moms would even share their opinions on various treatments and therapies as well and generate dialogue among one another.

Importantly, there are very few physicians actively posting clinically valid information about the rash, which could address many of the questions and concerns that these mothers pose online. Health care professionals, such as dermatologists and obstetricians, should be aware of this social media presence and consider increasing their influence on applications since a high number of patients turn to internet communities for support.

One suggestion we propose is for physicians to increase their social media presence by creating public professional accounts that display their credentials [13]. In this way, physicians can advertise their professional accounts to the existing clientele and reach patient populations beyond those that they personally serve. Once an account is established, posts can be made that combine informative graphics with educational text. Therefore, if physicians generate more content and use hashtags so that their posts are searchable, they could reach a larger audience interested in the topic. It might also behoove the physician to partner with bloggers, such as maternity bloggers in our case, in order to quickly gain visibility and reach a larger audience already seeking information and support [13].

The spread of misinformation has also become a topic of discussion in recent years as social media has become a dominant forum for peer conversation [13]. With the rise of social media as an arena for sharing, it has become apparent that perpetuation of incorrect medical statements may create mistrust and fear among patients [14]. Serious false information has been disseminated, such as the belief that vaccines cause autism, because physicians abuse the trust built. It is the duty of physicians, including physicians participating in online discourse, to ensure that facts are checked. Physicians are in a particularly unique position to create posts containing evidence-based information while also being key opinion leaders.

In a time where we have immediate access to any information through the internet, misinformation is rampant and physicians work hard to dispel concerns that patients bring in with them to the examination room. We suggest that clinicians build up their social media presence to offer legitimate responses and medical information to patients looking for quick answers before their next doctor’s appointment.

## Abbreviations

PUPPP: pruritic urticarial papules and plaques of pregnancy

## References

[1] Lehrhoff S, Pomeranz MK (2013). Specific dermatoses of pregnancy and their treatment. Dermatol Ther.

[2] Zhu P, Fung A, Woo BKP (2020). Consumer Preference of Products for the Prevention and Treatment of Stretch Marks: Systematic Product Search. JMIR Dermatol.

[3] Aronson IK, Bond S, Fiedler VC, Vomvouras S, Gruber D, Ruiz C (1998). Pruritic urticarial papules and plaques of pregnancy: Clinical and immunopathologic observations in 57 patients. Journal of the American Academy of Dermatology.

[4] Matz H, Orion E, Wolf R (2006). Pruritic urticarial papules and plaques of pregnancy: polymorphic eruption of pregnancy (PUPPP). Clin Dermatol.

[5] Zhao Y, Zhang J (2017). Consumer health information seeking in social media: a literature review. Health Info Libr J.

[6] Baker B, Yang I (2018). Social media as social support in pregnancy and the postpartum. Sex Reprod Healthc.

[7] Lam NH, Woo BK (2020). Efficacy of Instagram in Promoting Psychoeducation in the Chinese-Speaking Population. Health Equity.

[8] Sherman LE, Payton AA, Hernandez LM, Greenfield PM, Dapretto M (2016). The Power of the Like in Adolescence: Effects of Peer Influence on Neural and Behavioral Responses to Social Media. Psychol Sci.

[9] (2019). Social Media Fact Sheet. Pew Research Center for Internet and Technology.

[10] Martin JA, Hamilton BE, Osterman MJK, Driscoll AK (2019). Births: Final Data for 2018. Natl Vital Stat Rep.

[11] Chong KPL, Guo JZ, Deng X, Woo BKP (2020). Consumer Perceptions of Wearable Technology Devices: Retrospective Review and Analysis. JMIR Mhealth Uhealth.

[12] Chung S, Woo BK (2020). Using Consumer Perceptions of a Voice-Activated Speaker Device as an Educational Tool. JMIR Med Educ.

[13] Trethewey SP (2020). Strategies to combat medical misinformation on social media. Postgrad Med J.

[14] Larson HJ (2018). The biggest pandemic risk? Viral misinformation. Nature.

